# BAK and NOXA Are Critical Determinants of Mitochondrial Apoptosis Induced by Bortezomib in Mesothelioma

**DOI:** 10.1371/journal.pone.0065489

**Published:** 2013-06-07

**Authors:** Sara Busacca, Alex D. Chacko, Astero Klabatsa, Kenneth Arthur, Michael Sheaff, Vignesh K. Gunasekharan, Julia J. Gorski, Mohamed El-Tanani, V. Courtney Broaddus, Giovanni Gaudino, Dean A. Fennell

**Affiliations:** 1 Department of Cancer Studies and Molecular Medicine, University of Leicester, Leicester, United Kingdom; 2 Centre for Cancer Research and Cell Biology, Queen’s University of Belfast, Belfast, Northern Ireland; 3 Division of Cancer Studies, Department of Research Oncology, King’s College London, London, United Kingdom; 4 Department of Cellular Pathology, Barts and the London NHS Trust, London, United Kingdom; 5 Department of Microbiology-Immunology, The Feinberg School of Medicine, Northwestern University, Chicago, Illinois, United States of America; 6 Lung Biology Centre, San Francisco General Hospital, University of California San Francisco, San Francisco, California, United States of America; 7 University of Hawaii Cancer Center, Honolulu, Hawaii, United States of America; University of Illinois at Chicago, United States of America

## Abstract

Based on promising preclinical efficacy associated with the 20S proteasome inhibitor bortezomib in malignant pleural mesothelioma (MPM), two phase II clinical trials have been initiated (EORTC 08052 and ICORG 05–10). However, the potential mechanisms underlying resistance to this targeted drug in MPM are still unknown. Functional genetic analyses were conducted to determine the key mitochondrial apoptotic regulators required for bortezomib sensitivity and to establish how their dysregulation may confer resistance. The multidomain proapoptotic protein BAK, but not its orthologue BAX, was found to be essential for bortezomib-induced apoptosis in MPM cell lines. Immunohistochemistry was performed on tissues from the ICORG-05 phase II trial and a TMA of archived mesotheliomas. Loss of BAK was found in 39% of specimens and loss of both BAX/BAK in 37% of samples. However, MPM tissues from patients who failed to respond to bortezomib and MPM cell lines selected for resistance to bortezomib conserved BAK expression. In contrast, c-Myc dependent transactivation of NOXA was abrogated in the resistant cell lines. In summary, the block of mitochondrial apoptosis is a limiting factor for achieving efficacy of bortezomib in MPM, and the observed loss of BAK expression or NOXA transactivation may be relevant mechanisms of resistance in the clinic.

## Introduction

Malignant pleural mesothelioma (MPM) is an aggressive cancer caused by exposure to asbestos. It is increasing in incidence worldwide however there is a paucity of effective therapy [1]. Pemetrexed or raltitrexed when combined with cisplatin have been shown to lead to modest improvements in overall survival [2], [3]. However, patients universally relapse; following which, there is no agreed standard of care. MPM is a highly drug resistant cancer, and this is correlated with apoptosis resistance [4]. There is a pressing need for new, more effective therapies, particularly where there is an unmet clinical need after first-line chemotherapy.

The proteasome inhibitor bortezomib has shown promising activity in preclinical models both in vitro and in vivo [5], [6], which has led to initiation of clinical trials evaluating the effect of bortezomib alone [7] or in combination with cisplatin or oxaliplatin (www.clinicaltrials.gov). However our data from Phase II study of bortezomib activity as monotherapy in an unselected population of MPM patients showed only minimal (5%) response rate, implicating inherent resistance [7].

In contrast to hematopoietic malignancies, the poor response of solid tumours to bortezomib treatment appears to be due to the existence of both primary and acquired resistance [8]. Several mechanisms of resistance have been proposed, including mutations in the proteasome subunits and alteration in their expression levels [9]–[14], increases in the efficiency of alternative mechanisms of protein degradation such as the lysosomal system, the ER-associated protein degradation (ERAD) [15], and aggresome formation [16]. Bortezomib is an activator of the mitochondrial apoptosis pathway, and as such, defects in this signalling pathway could confer resistance [17].

Here, we show that specific components of the mitochondrial signalling pathway are lost or dysregulated in MPM, and can directly cause bortezomib resistance.

## Materials and Methods

### Reagents and Antibodies

Bortezomib was courtesy of Millenium; MG132 was purchased from Sigma-Aldrich, (St. Louis, MO). Antibodies against c-Myc, BAX and BAK were from Cell Signaling (Danvers, MA, USA), anti-PARP from Alexis (Nottingham, UK), anti-NOXA from Calbiochem (Gibbstown, NY), GAPDH and β-Tubulin from Abcam (Cambridge, UK). Secondary antibodies were: goat anti-rabbit HRP (DAKO, Glostrup, Denmark), donkey anti-mouse HRP (GE Healthcare).

### Cell Lines

REN [18] (kindly provided by Dr. S.M. Albelda, University of Pennsylvania, Philadelphia, USA), were grown in Nutrient mixture F12 Ham (Invitrogen, Carlsbad, CA), L-Glutamine, 10% (FBS) Foetal Bovine Serum (PAA) and penicillin/streptomycin (Gibco). JU77 [19], and Wild type (WT) and BAX/BAK double knockout (DKO) mouse embryonic fibroblasts (MEFs) [20] (kind gift from Dr. Scott Oakes,University of California, San Francisco, USA) were grown in RPMI Medium 1640, L-Glutamine and 10% FBS. Bortezomib resistant cells (REN^BZR^, JU77^BZR^) were generated by increasing exposure to bortezomib. Generation of c-Myc shRNA expressing stable clones employed retroviral transduction using 4×10^5^ Phoenix Ampho cells [21] (kindly provided by P. Mullan, Queen’s University of Belfast, Northern Ireland). Cells were and transfected with pRetroSuper c-Myc shRNA (Addgene) or pRetroSuper scrambled using GeneJuice (Novagen, Madison, Wisconsin). 48 h post transfection, the media was filtered and added to REN cells for 3 h. Cells were then subjected to puromycin (Calbiochem) (4 µg/mL) selection.

### Measurement of Cell Viability and Apoptosis

Cell viability was assessed by a Vialight Plus kit (Lonza, Basel, Switzerland). For the caspase-3 luminescence assay, cells were analysed by using a Caspase-Glo 3/7 Assay (Promega, Southampton, Hampshire).

### Protein Extraction and Immunoblotting

Cells were lysed in RIPA buffer containing protease inhibitors (Roche, Burgess Hill, UK). Cell lysates were separated on SDS-PAGE denaturing gels, transferred to nitrocellulose membranes, and blocked in 5% milk-PBS-0.1% tween. Membranes were probed with primary antibodies diluted in 5% milk-PBS-0.1% tween at 4°C overnight. Signal detection was performed with the ECL-plus chemiluminescent system (GE Healthcare, Amersham, UK).

### siRNA Transfections

Non-silencing control (NT), BAX, BAK, and NOXA targeting siRNAs were obtained from Qiagen. siRNA (50 nM for BAX and BAK, 20 nM for NOXA) transfections were performed using the RNAiMAX transfection reagent (Invitrogen) according to manufacturer’s instructions.

### BAX and BAK Overexpression

BAX/BAK DKO cells were transiently transfected with GST-tagged BAX and BAK (pEGFP-C3 vector), using X-tremeGENE transfection reagent (Roche) according to manufacturer’s instructions.

### Tumour Samples

BAX and BAK protein expression was assessed by immunohistochemistry (IHC) on two sets of samples. 16 tissues were from ICORG-05 Study [7]. The second set comprised a TMA of 100 archived mesotheliomas; however partial of full loss of 30% TMA cores has been observed during the staining. Appropriate ethical approval was obtained from the local research ethics committees to carry out this work (Ireland: SJH/AMNCH (The St James’s Hospital & Adelaide & Meath Hospital incorporating the National Children’s Hospital) Research Ethics Committee; Netherlands: Ethics Committee of NKI-AVL (Nederlands Kanker Instituut - Antoni van Leeuwenhoek Ziekenhuis); Belgium: Ethics Committee of the University Hospital Ghent; United Kingdom: Belfast Central Research, OREC (Office of research ethics committees) UK.

### Immunohistochemistry and Scoring

Immunohistochemistry for the samples from the ICORG-05 study was performed within the Tissue Core Technology Unit at the Centre for Cancer Research and Cell Biology and sections were then scanned in the Queen’s University of Belfast Bioimaging Unit. BAK primary antibody was used at a 1∶800 dilution; BAX antibody was used at a 1∶50 dilution. IHC scoring of tissue slides from the Phase II trial was carried out through the PathXL™ TMA Toolbox (i-Path Diagnostics Ltd, Belfast, UK). The TMA was stained and scored in the Pathology Core Facility, Department of pathology, Bart’s and the London NHS Trust.

The staining results were semi-quantitatively assessed by two individual examiners. Tumours were graded for expression of BAX and BAK as follows: 0 = no cells stained, 1 = <25% cells positive (light staining), 2 = 25–75% cells positive (moderate staining) 3 = >75% cells positive (strong staining). In case of discrepancy between the two examiners, a result was obtained by consensus while reviewing the slides using a double-headed microscope. Survival data were available for 30 out of 70 patients; therefore the analysis has been carried out on this unselected population only. The survival analysis was performed by using Kaplan-Meier estimation and significance was measured by the log-rank test.

The adopted statistics software was SPSS17.0 (Chicago, IL, USA).

### Mitochondria Isolation

Cells were washed in Mitochondrial Isolation Buffer (200 mM Mannitol, 70 mM Sucrose, 1 mM EGTA, 10 mM HEPES, 0.5 mg/ml BSA, pH7.4). Mitochondria were then isolated by dounce homogenization followed by centrifugation at 800×g for 10 minutes at 4°C to remove debris and heavy membranes, then by centrifugation at 10,000×g for 10 minutes at 4°C. The mitochondrial-free cytosolic fraction was used for Western blot analysis [22].

### Real Time Quantitative RT-PCR

Total RNA was extracted using an RNeasy Plus mini kit (Qiagen Valencia, CA, USA) according to manufacturer’s instructions. Quality control was performed by Phalanx Biotech Group (Palo Alto, CA, USA). Reverse transcription was performed with M-MLV Reverse transcriptase (Invitrogen). Real-Time PCR was carried out using Power SYBR® Green PCR Master Mix (Applied Biosystem).

### c-Myc Reporter Assay

c-Myc reporter assay was performed using Cignal Reporter Assay Kits (SABiosciences, Frederick, MD). Transfections were carried out by using the Lipofectamine2000 transfection reagent (Invitrogen). Luciferase activity was measured using the dual-luciferase reporter assay system (Promega).

### Chromatin Immunoprecipitation

Chromatin immunoprecipitation assays were performed as previously described [23]. The antibodies used for immunoprecipitation were: TBP antibody, HA-probe (Y11), anti-c-Myc (N262), from Santa Cruz and rabbit IgG from Dako. PCR amplification was performed on the purified DNA using the primers for MYC-BS III [24].

### Statistical Analysis

Dose-response curves were fitted using non-linear regression (GraphPad Prism version 4.0, GraphPad Software, Inc. LaJolla, CA, USA).

One or two-way analysis of variance was used to evaluate statistical significance and a Bonferroni post-test was performed. A p value less than 0.05 was considered significant.

## Results

### BAK is an Essential Regulator of Bortezomib-induced Apoptosis in MPM Cells

**Figure 1:**
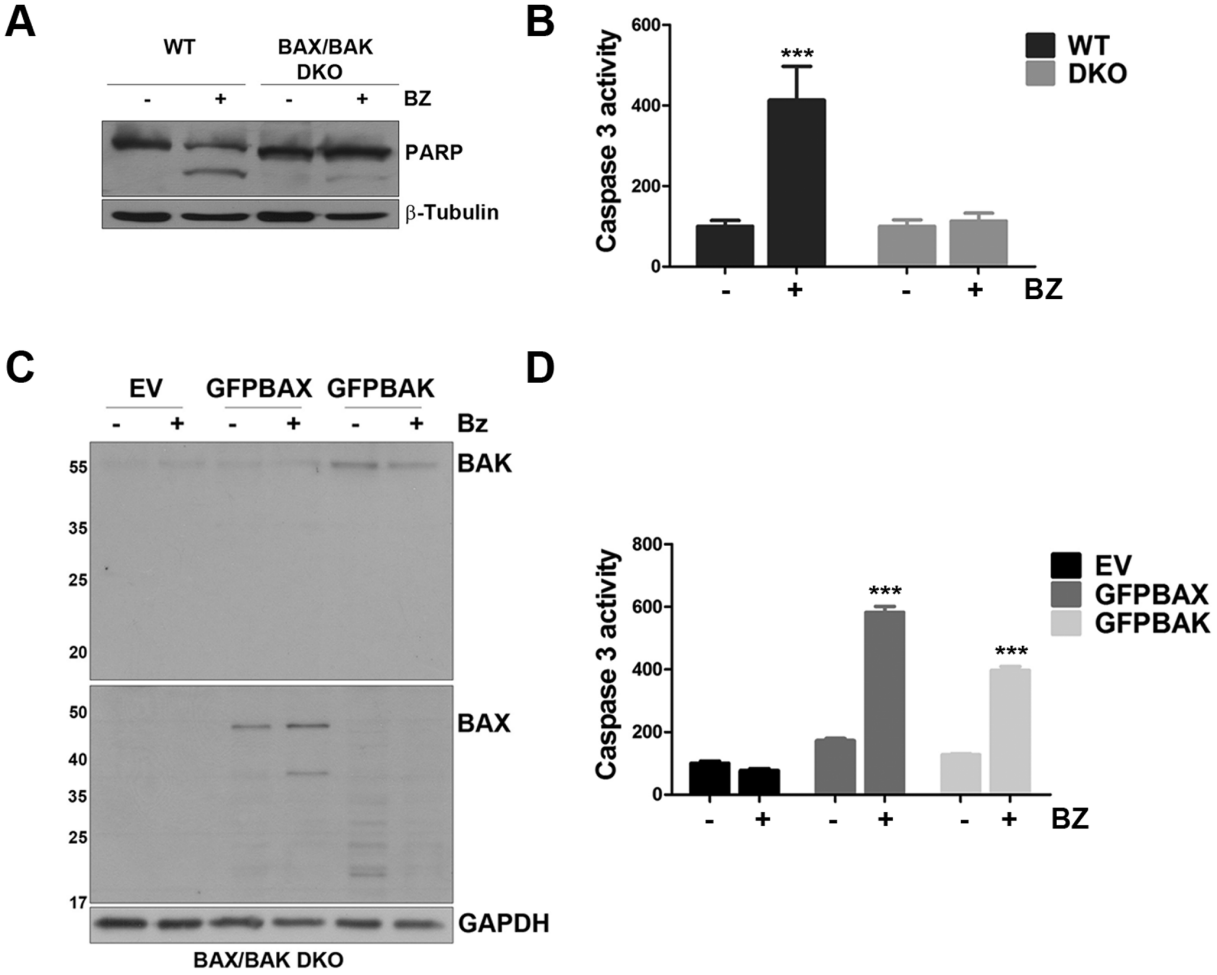
In MEF cells, BAX and BAK expression regulates bortezomib activity. A) WT MEF and BAX/BAK DKO MEF cells were treated with bortezomib 10 nM for 24 h. PARP cleavage was measured by western blot. B) Caspase3 activity was assessed by luminescence assay. Data were normalized to untreated control (WT: p<0.0001; DKO n.s.). C) BAX and BAK were transiently overexpressed in DKO cells and 24 h post transfection cells were treated with bortezomib 10 nM for a further 24 hours. BAX and BAK expression were then analysed by western blot. D) Caspase 3 activation after bortezomib treatment was also analysed by luminescence assay. Data were normalized to untreated control (EV: n.s.; GSTBAX: p = <0.0001 GSTBAK: p = <0.0001).

Bortezomib induced apoptosis in wild type mouse embryonic fibroblasts (WT MEF), but this effect was dramatically reduced in cells with homozygous deletion of BAX and BAK (BAX/BAK DKO MEF), as evidenced by PARP cleavage (**Figure 1A**) and caspase 3 activation (4.13 and 1.13 fold increase in WT and DKO respectively) (**Figure 1B**). Apoptosis induction measured by caspase 3 activation, was restored by knocking-in BAK or BAX (EV: 0.77, GST-BAX: 3.48 and GST-BAK: 3.04 fold increase) (**Figure 1C, D**), suggesting that both BAX and BAK mediate bortezomib-induced apoptosis.

**Figure 2:**
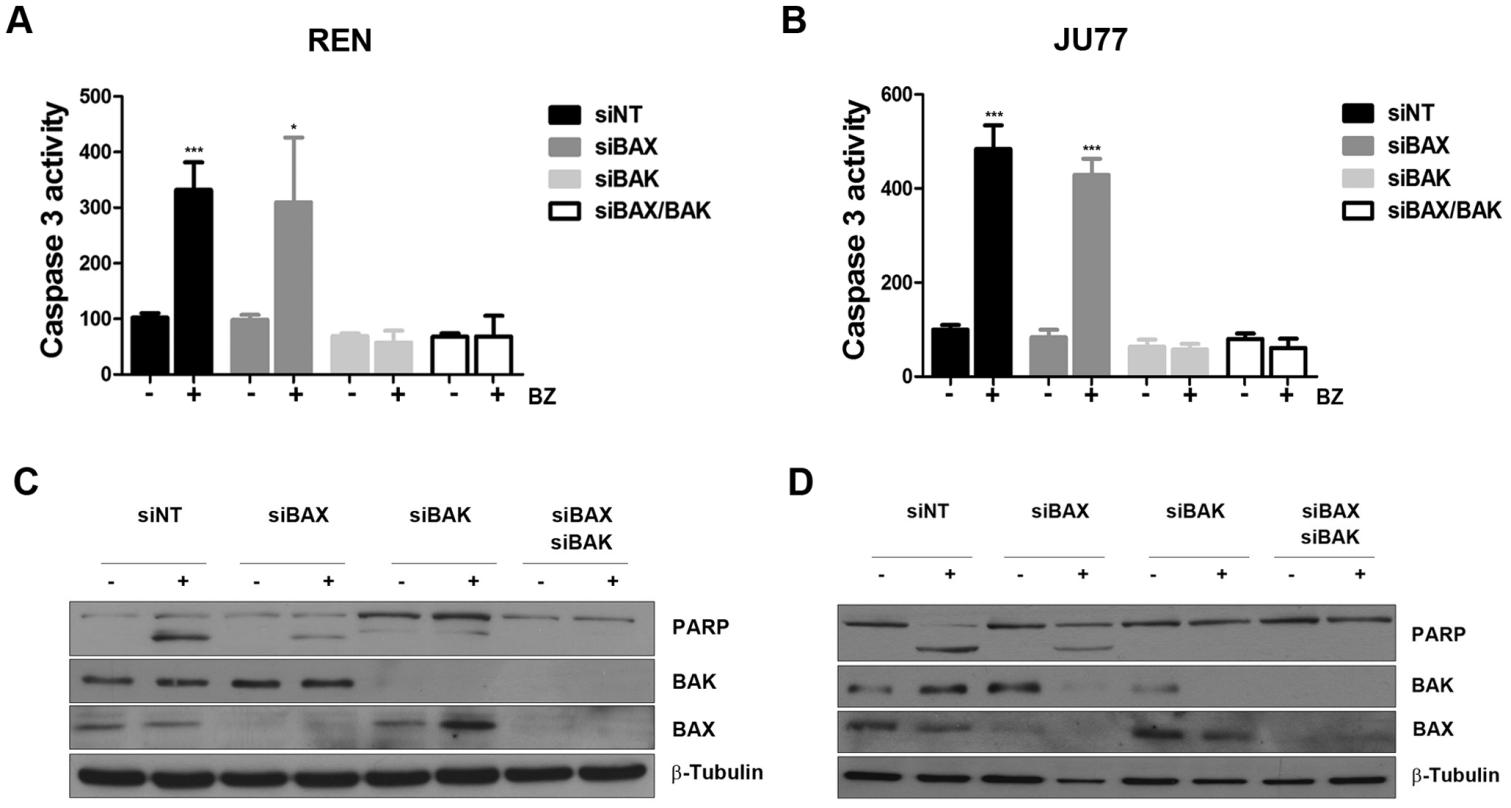
In MPM cell lines, dowregulation of BAK induces resistance to bortezomib-induced apoptosis. A) REN and B) JU77 cells were transfected with siNT, siBAX, siBAK and the combination of siBAX and siBAK. 24 h following transfection, cells were treated with a concentration of bortezomib equal to the IC_50_ calculated for each cell line and caspase3 activity measured. Data were normalized to NT untreated control (REN: siNT p = 0.0003 siBAX p = 0.0159; siBAK n.s. siBAXsiBAK n.s.; JU77: siNT p = 0.0002 siBAX p = 0.0002; siBAK n.s.; siBAXsiBAK n.s.). C) BAX and BAK expression and PARP cleavage were confirmed by western blot analysis in REN and D) JU77 cells.

We then tested for the individual and combined contribution of BAX and BAK by using RNA interference to silence BAX or BAK or both in MPM cell lines (REN and JU77). Here in contrast to our findings using MEFs, we found that silencing of BAX alone did not reduced sensitivity to bortezomib. However, BAK proved to be important; BAK and BAX/BAK silencing significantly reduced bortezomib-induced caspase 3 activity in both REN (siNT: 3.25, siBAX: 3.14, siBAK: 0.83 and siBAX/BAK: 1.01 fold increase) (**Figure 2A**) and JU77 (siNT: 4.8, siBAX: 5.13, siBAK: 0.9 and siBAX/BAK: 0.76 fold increase) (**Figure 2B**). Similarly, also PARP cleavage was reduced only by the expression of BAK, but not of BAX in both REN (**Figure 2C**) and JU77 (**Figure 2D**).

### BAK is Lost in Primary Mesothelioma

**Figure 3:**
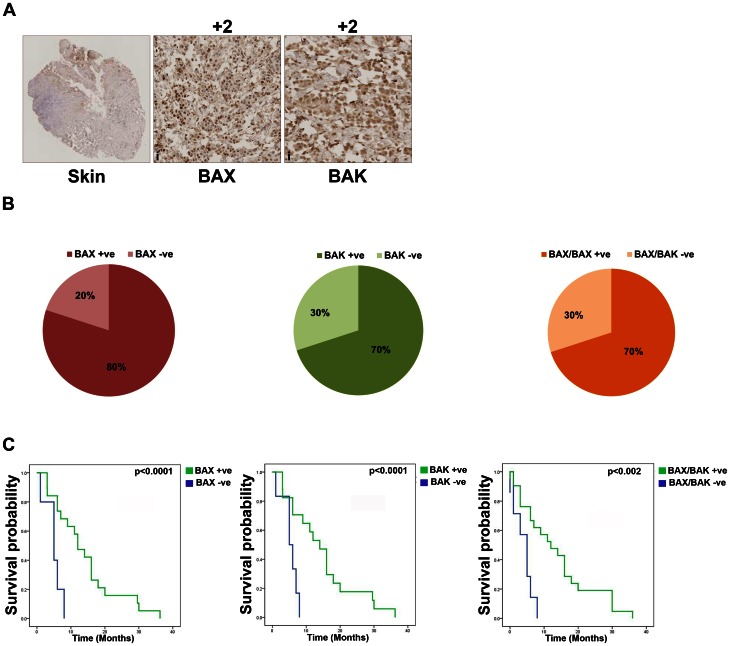
Immunohistochemical analysis of BAX and BAK expression in malignant mesothelioma patients. A) Representative image of normal tissue control and positive tissues stained for BAX and BAK by immunohistochemisty. B) Pie-charts representing the frequency of BAX, BAK and BAX-BAK negativity in previously treated 30 mesothelioma patients. C) Kaplan Meier curves correlating BAX, BAK, and double BAX/BAK expression respectively with survival in the total of 30 previously-treated patients.

We used an immunohistochemistry-based approach to measure the expression of BAX and BAK in two different cohorts of MPM patients (**Figure 3A**). In the first cohort, out of 69 cases assessed for BAK expression, 43 were positive (62.3%) and 26 were negative (37.7%). The staining was associated mainly with moderate or strong intensity in both cases. The expression of BAX could be assessed in 70 cases overall the TMA of which 44 cases were positive (62.9%) and 26 were negative (37.1%). The loss of both BAX and BAK expression was also analysed in 70 cases of which 44 were positive for both proteins (62.9%) and 26 were negative for both proteins (37.1%). Thirty of these patients were previously treated and survival data were available; of these patients the 80% presented positive staining for BAX, 70% were positive for BAK and 70% were positive for both BAX and BAK (**Figure 3B**). Survival analysis revealed that in BAK positive patients the overall median survival was 18 versus 6 months in negative patients (HR = 0.518, 95% CI = 0.36–0.75). In the case of BAX, its expression was associated with a survival of 16 months, compared to 5 months of BAX negative patients (HR = 0.42, 95% CI = 0.29–0.62). The overall median survival was 18 months in BAX/BAK positive samples and 5 months in BAX/BAK negative samples (HR = 0.197, 95% CI = 0.08– 0.49) (**Figure 3** **C**).

A second cohort consisted of 16 specimens obtained from MPM patients treated with bortezomib in a phase II trial [7]. BAX expression was absent in 50% of the tumours, whereas BAK was expressed in all the samples (100%). Notably, only one patient (sample i) of this set had stable disease [7] and the corresponding tissue was positive for both BAX and BAK staining (**Table 1**).

**Table 1:** Scoring of samples from ICORG-05 Phase II Trial [7].

	Staining intensity			Staining intensity	
Sample	BAX	BAK	Sample	BAX	BAK
**a**	1	1	**i**	2	2
**b**	0	1	**j**	0	2
**c**	1	2	**k**	0	1
**d**	0	1	**l**	1	1
**e**	0	1	**m**	0	2
**f**	1	1	**n**	1	1
**g**	0	1	**o**	2	3
**h**	0	3	**p**	1	1

0 = No cells stained 1 = <25% cells stained 2 = 26–75% cells stained 3 = >75% cells stained.

### Neither BAX or BAK Expression are Altered in Mesothelioma Cell Lines Selected for Bortezomib Resistance

**Figure 4:**
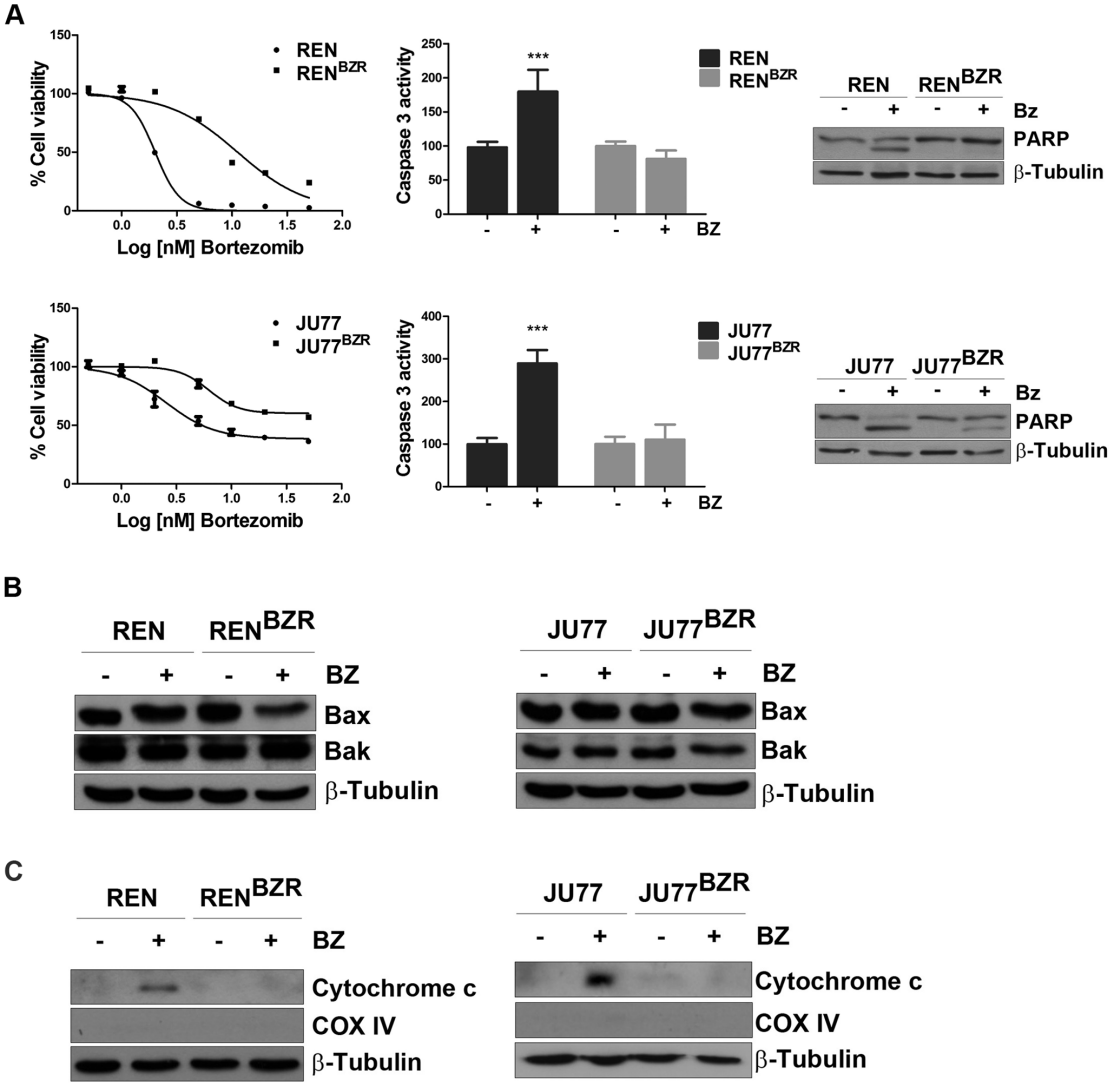
Generation and characterization of mesothelioma bortezomib-resistant cell lines. A) REN and JU77 selected for resistance after exposure to increasing doses of bortezomib were tested for cell viability after 24 h treatment with bortezomib at concentrations ranging from 0.5 nM to 50 nM and compared to parental cells. REN/REN^BZR^ and JU77/JU77^BZR^ cells were treated for 24 h with bortezomib 5 nM and 10 nM, respectively. PARP cleavage induced by bortezomib was analysed by western blot and caspase3 activity was measured by luminescence assay. Data were normalized to untreated control (REN: <0.0001; REN^BZR^: n.s.; JU77: p = 0.0002; JU77^BZR^: n.s.). B) Expression of BAX and BAK was investigated in parental and resistant cells pre- and after 6 h treatment with bortezomib 5 nM and 10 nM in REN/REN^BZR^ and JU77/JU77^BZR^ respectively. C) Cytochrome C release was assessed after 24 h treatment with bortezomib (5 nM and 10 nM in REN/REN^BZR^ and JU77/JU77^BZR^, respectively). Mitochondrial-free cytosolic fraction has been used for western blot analysis.

Both REN and JU77 cell lines were exposed to increasing concentrations of bortezomib leading to selection of two isogenic resistant cell lines (REN^BZR^, JU77^BZR^). REN^ BZR^ and JU77^ BZR^ cells exhibited 6-fold and 7-fold resistance compared to parental cells (IC_50_ 12 nM and 50 nM respectively) (**Figure 4A, left panel** **s**). Resistance was accompanied by a significant reduction in caspase 3 activity (REN: 1.83, REN^BZR^: 0.8, JU77∶2.9, JU77^BZR^: 1.1 fold increased) (**Figure 4A** **, middle panels**) and reduced PARP cleavage in both resistant cell lines compared to parental cells (**Figure 4A, right panel** **s**). Basal expression of both BAX and BAK was not significantly different in resistant cells compared to parental cell lines after 6 h (**Figure 4B**). Finally, cytochrome C release upon bortezomib treatment was also investigated; cytosolic levels of Cytochrome C in a cytoplasmic mitochondria-free fraction were detected in parental cell lines only, but not in the two resistant cell lines **(** **Figure 4C** **)**.

### The BH3-only Protein NOXA is Critical for Bortezomib-induced Apoptosis

**Figure 5:**
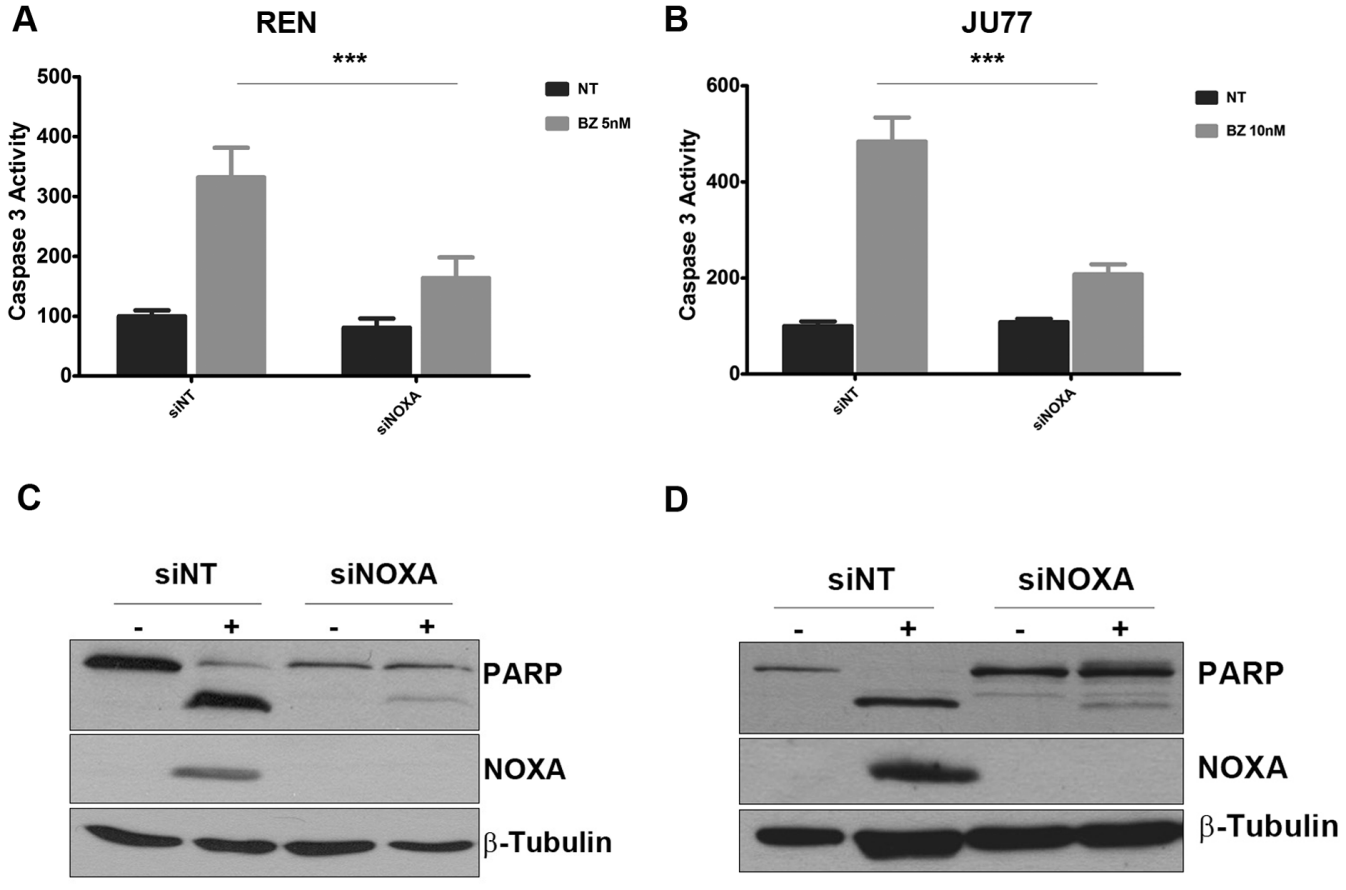
The BH3-only protein NOXA is essential for bortezomib-induced apoptosis. A) REN and B) JU77 cells were transfected with siRNA sequences targeting the BH3-only protein NOXA. 24 h following transfection, cells were treated with a concentration of bortezomib equal to the IC_50_ calculated for each cell line and caspase3 activity measured. Data were normalized to NT untreated control. C) NOXA expression level and PARP cleavage were assessed by western blot analysis in REN and D) JU77 cells.

Upregulation of NOXA has been implicated as a regulator of bortezomib induced apoptosis specifically in tumour cells [17]. After NOXA was silenced using RNA interference, bortezomib induced caspase 3 activity was significantly inhibited in REN (**Figure 5A**) and JU77 transfected cells (**Figure 5B**). These data were confirmed by western blot showing a significant decrease in PARP cleavage induced by bortezomib in both REN (**Figure 5C**) and JU77 transfected cells (**Figure 5D**).

### Bortezomib Resistant Cells Fail to Activate Transcription of NOXA

**Figure 6:**
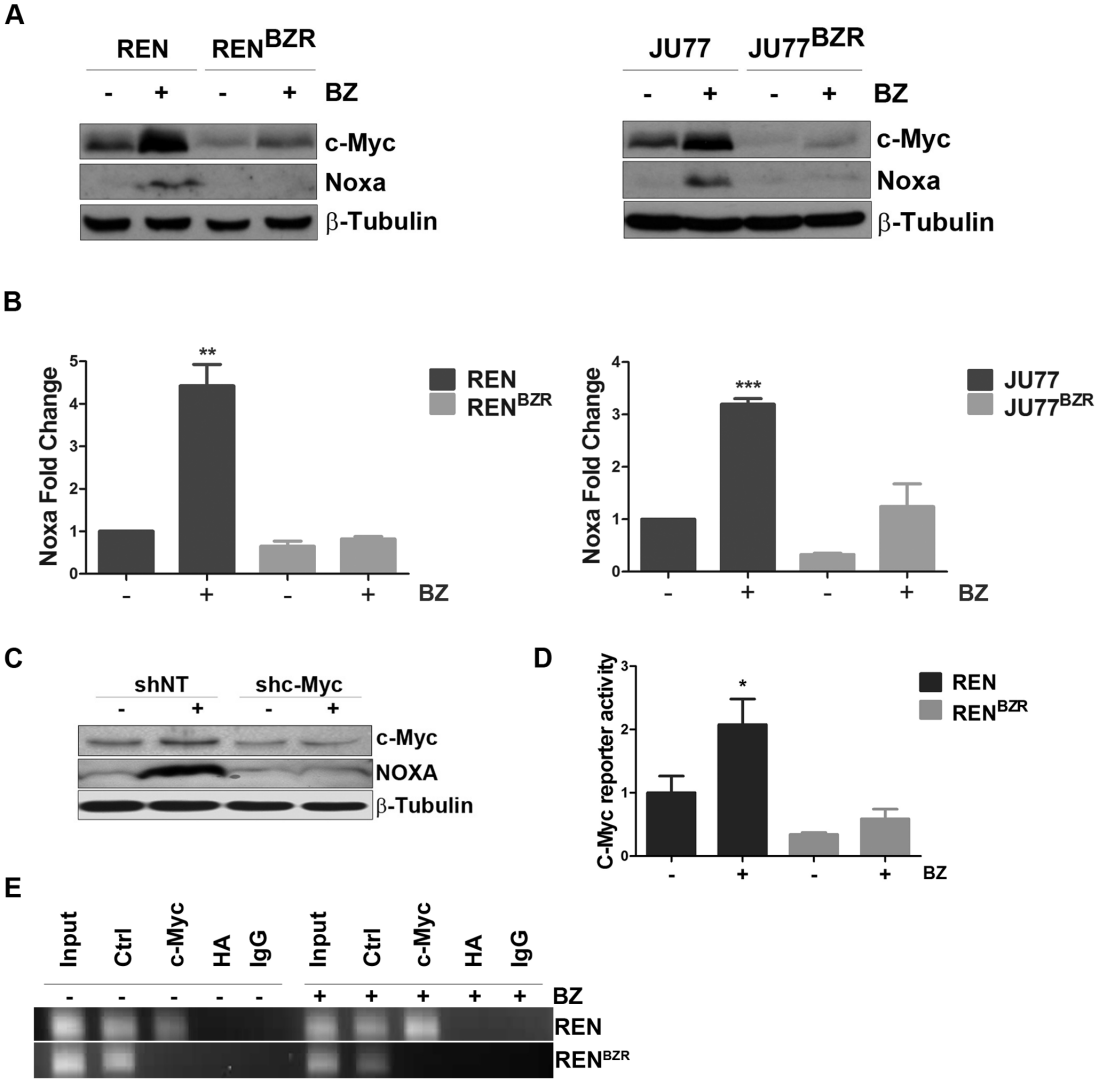
Selection for resistance to bortezomib abrogates c-Myc activity and NOXA expression. A) The expression of NOXA and c-Myc was evaluated in by western blotting in REN/REN^BZR^ and JU77/JU77^BZR^ cells. Cells were left untreated or exposed to bortezomib 5 nM or 10 nM respectively for 6 hours. B) NOXA mRNA expression was evaluated by qRT-PCR on RNA extracted from parental and resistant cells treated for 6 h with 5 nM (REN/REN^BZR^) or 10 nM (JU77/JU77^BZR^) bortezomib. Data were normalized to untreated control (REN: p = 0.00241; REN^BZR^ n.s.; JU77: p = 0.0001; JU77^BZR^ n.s.) C) REN^shNT^ and REN^shc-Myc^ cells were generated by RNAi and NOXA induction was analysed by western blot after 24 h treatment with 5 nM bortezomib. D) c-Myc activity was measured by a reporter assay in REN and REN^BZR^ treated for 24 h with 5 nM bortezomib. Data were normalized to untreated control (REN: p = 0.0438; REN^BZR^ n.s.). E) The binding of c-Myc to the Noxa promoter was evaluated by ChIP in REN and REN^BZR^ treated for 24 h with 5 nM bortezomib.

The upregulation of NOXA protein expression following bortezomib was significantly reduced in resistant cells compared to parental cells (**Figure 6A**). Analysis of NOXA mRNA level revealed significant transcriptional upregulation of NOXA after bortezomib treatment in parental cells. This was not reflected in the resistant cell lines where bortezomib induced very little or no upregulation of NOXA (REN: 4.42; REN^BZR^: 0.82; JU77∶3.19; JU77^BZR^: 1.24 fold increase, respectively) **(** **Figure 6B** **).**

C-Myc is a transcriptional activator of NOXA [24]. To evaluate the role of this transcription factor in MPM cells, REN clones stably expressing shRNA targeting c-Myc (REN^sh/Myc^), or scrambled control shRNA (REN^sh/scr^), were generated. In REN^sh/Myc^ cells following bortezomib treatment, the upregulation of NOXA protein was completely abolished following bortezomib in REN^sh/Myc^ cells (**Figure 6C**).

In bortezomib resistant MPM cells, c-Myc protein expression was lower at baseline compared to parental cells and was unaffected by exposure to bortezomib (**Figure 6A**). Consistently, the basal transcriptional activity of c-Myc in resistant cells evaluated by a luciferase reporter assay was significantly reduced compared to parental cells. Following bortezomib treatment c-Myc activity was significantly increased in REN but not in REN^BZR^ cells (REN: 2.08; REN^BZR^: 1.7 fold increase, respectively) (**Figure 6D**).

Chromatin immunoprecipitation revealed the interaction of c-Myc with the promoter for NOXA in REN parental cells, which increased after treatment with bortezomib. However, no interaction was observed in REN^BZR^ cells, even after treatment with bortezomib (**Figure 6E**).

## Discussion

Bortezomib exhibits significant preclinical activity in several solid tumour cell lines and animal models including MPM [5], [6]. However, this efficacy has not been successfully translated into the clinic due to either primary or acquired resistance [7], [25]–[27]. Here we demonstrate that reconstitution of BAX or BAK in mouse BAX/BAK DKO fibroblasts is sufficient to restore sensitivity to bortezomib. In MPM cells the silencing of BAK dramatically reduced response to bortezomib, however downregulation of BAX alone was not sufficient to impact the response to bortezomib. Thus, although BAX and BAK appear to be functionally redundant in MEFs, this does not appear to be the case in MPM cells, where BAX was not able to reconstitute loss of BAK. This might be due to the known differential regulation by prosurvival proteins, such as Mcl-1 and Bcl-xL [28]–[30]. Both Bcl-xL and Mcl-1 are highly expressed in MPM and may restrain BAK constitutively [31]. Moreover, BAX and BAK expression data in our resistant cell lines show that these proteins are not altered in a context of “acquired resistance” after prolonged exposure to the drug. This suggests that the selective pressure may not be enough to lead to loss of expression of proteins that have essential housekeeping roles, such as mitochondrial fusion and fission. However, in a clinical setting we observed de novo lack of BAX and BAK protein expression that can correlate with primary resistance.

The expression of BAX and BAK has been previously investigated in mesothelioma samples and 24% loss of BAK and 42% loss of BAX expression were found, but no correlation with histology was reported [31]. Conversely, another group showed 100% expression of BAX in the MPM specimens analysed [32]. In our population of unselected MPM samples loss of both BAX and BAK was observed and it was correlated with clinical outcome implicating a prognostic significance of defective mitochondrial apoptosis. However, Cox regression analysis was only possible for histology, age and sex and no association between those and BAX/BAK expression could be seen. This is due to the low number of BAX/BAK positive cases with survival data not allowing statistical analysis. Therefore, the prognostic value of BAX/BAK levels associated with other common prognostic factors requires further analysis in a larger cohort with available clinical data.

Apoptosis block is a hallmark of cancer and may contribute to aggressive tumour progression in this sub-population of patients with MPM, as well as potentially conferring drug resistance [33].

Bortezomib upregulates the BH3-only protein and Mcl-1 inhibitor NOXA, at both the protein and mRNA level after 6 hours from exposure [22], [34], [35]; The Mcl-1 anti-apoptotic protein inhibits apoptosis by sequestering BAK from activating the mitochondrial outer membrane permeabilization [30]. It has been demonstrated that NOXA can displace BAK from Mcl-1 and can also promote Mcl-1 degradation trough the proteasome system [30]. Here we show that NOXA is downregulated in both resistant cell lines; this may explain why NOXA or BAK silencing recapitulate the resistant phenotype. Moreover, treatment with bortezomib failed to induce NOXA upregulation at both protein and mRNA level in resistant cells.

Noxa is commonly described as a p53 target gene as it contains p53 response elements on its promoter and it has also been reported as a key mediator of p53-driven apoptosis [36]. However, it has been shown that in different cellular systems the up-regulation of Noxa at both protein and mRNA level induced by bortezomib can also occur through p53-independent mechanisms [22], [34], [37], [38], such as myc transcriptional regulation [24]. Indeed, it was demonstrated that c-Myc can bind the Noxa promoter and regulate its transcription [24], [39]. As shown in Figure 6, Noxa overexpression occurs in REN cells upon treatment with bortezomib. The transcriptional mechanism involved in REN cells must be p53-independent because REN cells are known to contain a rearranged p53 gene and lack expression of p53 protein [40]. We thus suggest the c-Myc is the only driver of Noxa expression in this contest.

The induction of NOXA and the subsequent activation of the mitochondrial apoptosis pathway by bortezomib has been reported not to correlate linearly with c-Myc protein or mRNA expression [24], but being dependent on c-Myc activity in binding the Noxa promoter. However, no data are available to date on c-Myc dysregulation in bortezomib resistance cells. Here we show that resistant cells express a lower level of c-Myc protein, compared to parental cells and that c-Myc is dysfunctional in the resistant setting. No binding at the Noxa promoter was detected in resistant cells and as expected it was not induced by bortezomib treatment. Our data support the thesis of c-Myc stabilisation induced by bortezomib (as shown by western blot), consequent increase in binding to the Noxa promoter (ChIP data) and final activation (increased reporter activity).

These events may occur in cooperation with proteasome function in regulating histone acetylases [41], chromatin modulating proteins, basal transcriptional factors and DNA methyltransferase [42] which play an important role in c-Myc transcription regulation [43]–[45].

Alternative mechanisms of resistance to bortezomib have been proposed, in fact, increasing evidence in haematological tumours support the importance of the expression levels of proteasome subunits and their composition. Mutation of PSMB5 has been shown to be a cause of bortezomib resistance [10], [11], [13], [46], however G322A or C326T mutations were not observed in our cells implicating an alternative gene alteration, to account for lack of inhibition. Moreover, in our system expression levels of the β1 subunit before or after treatment were not altered. Conversely the β2 subunit was decreased in resistant cells, supporting data which showed a correlation between resistance and expression levels of proteasome subunits [47]. Finally, the β5 subunit level was increased in REN^BZR^ cells compared to parental cells (data not shown).

These findings suggest that disruption of c-Myc-dependent NOXA mediated death signalling and BAK could play a potential role in resistance to bortezomib in the clinical setting, highlighting the putative role of BAK and NOXA as valid prognostic markers for bortezomib. However, our data from 16 patients enrolled in the Phase II clinical trial showed that NOXA was expressed in the tissues from all the MPM patients examined, including the one showing stable disease [7]. The number of samples included in this analysis was too small to allow a statistically reliable analysis; actually, we know that a portion of patients will be BAX and/or BAK negative as observed in a larger cohort (70 samples). Nevertheless these patients will still show resistance even were NOXA expression is observed. It is also possible that other resistance mechanisms are involved in the clinical setting as suggested from data available from the sanger database (http://www.cancerrxgene.org/) regarding genomics of proteasome inhibitors, such as bortezomib and MG132, sensitivity/resistance.

In summary, bortezomib requires functional BAK and NOXA to induce apoptosis in MPM cells. The loss of BAK expression occurring in a subset of patients with MPM may contribute to resistance to this drug in the clinical setting. However, dysregulation of NOXA transactivation may be an alternative mechanism as evidenced in MPM cells selected for resistance to bortezomib.
