# Correction: BAK and NOXA Are Critical Determinants of Mitochondrial Apoptosis Induced by Bortezomib in Mesothelioma

**DOI:** 10.1371/annotation/90879c69-ebc5-40b2-9926-4a00e4f9e055

**Published:** 2013-12-13

**Authors:** Sara Busacca, Alex D. Chacko, Astero Klabatsa, Kenneth Arthur, Michael Sheaff, Dario Barbone, Luciano Mutti, Vignesh K. Gunasekharan, Julia J. Gorski, Mohamed El-Tanani, V. Courtney Broaddus, Giovanni Gaudino, Dean A. Fennell

Dr. Dario Barbone was not included in the author byline. He should be listed as the sixth author and affiliated with Lung Biology Centre, SFGH, UCSF, San Francisco, CA, USA. The contributions of this author are as follows: Dr Barbone critically reviewed the paper together with Prof. Courtney Broaddus. He also participated in scientifically relevant discussion of the experiments.

Dr. Luciano Mutti was not included in the author byline. He should be listed as the seventh author and affiliated with Department of General Medicine, Vercelli and Borgosesia Hospitals, Vercelli, Italy. The contributions of this author are as follows: Dr Mutti actively and critically reviewed the paper.

